# 

**Published:** 1889-08

**Authors:** 


### CONTRIBUTORS TO VOLUME XXIX.

Banta, Rollin L., M. D. . . . . . . . . . . . . . . . . . . . . . Buffalo
Billings, Frank S., M. D. . . . . . . . . . . . . . . . . . . . . Chicago
Bradnack, F.. M. D. . . . . . . . . . . . . . . . . . . . . . . . Buffalo
Clark, Edward, M. D. . . . . . . . . . . . . . . . . . . . . . . Buffalo
Crego, Floyd S., M. D . . . . . . . . . . . . . . . . . . . . . . Buffalo
Cushing, Clinton, M. D. . . . . . . . . . . . . . . . . . . . . San Francisco
Da Costa, John C., M. D. . . . . . . . . . . . . . . . . . . . . Philadelphia
Dagenais, A., M. D. . . . . . . . . . . . . . . . . . . . . . . . Buffalo
Eliot, Llewellyn, M. D. . . . . . . . . . . . . . . . . . . . . Washington
Gross, Ferdinand H., M. D. . . . . . . . . . . . . . . . . . . . Philadelphia
Grosvenor, J. W., M. D. . . . . . . . . . . . . . . . . . . . . . Buffalo
Hayd, Herman E., M. D. . . . . . . . . . . . . . . . . . . . . . Buffalo
Henry, Frederick P., M. D. . . . . . . . . . . . . . . . . . . . Philadelphia
Howell, S. Y., M. D. . . . . . . . . . . . . . . . . . . . . . . . Buffalo
Hoffman, Joseph, M D. . . . . . . . . . . . . . . . . . . . . . Philadelphia
Hubbell, A. A., M. D. . . . . . . . . . . . . . . . . . . . . . . . Buffalo
King, A. F. A., M. D. . . . . . . . . . . . . . . . . . . . . . . Washington
King, Clarence, M. D. . . . . . . . . . . . . . . . . . . . . . Machias, N. Y.
Krauss, William C., M. D. . . . . . . . . . . . . . . . . . . . . Buffalo
Lothrop, Thos., M. D. . . . . . . . . . . . . . . . . . . . . . . . Buffalo
McMurtry, Lewis S., M. D. . . . . . . . . . . . . . . . . . . . . Louisville
Mann, Matthew D., M. D. . . . . . . . . . . . . . . . . . . . . . Buffalo
Massey, G. Betton, M. D. . . . . . . . . . . . . . . . . . . . . Philadelphia
Miller, John A., Ph. D. . . . . . . . . . . . . . . . . . . . . . . Buffalo
Mink, Arthur E., M. D. . . . . . . . . . . . . . . . . . . . . . . Rochester
Mulligan, E. W., M. D. . . . . . . . . . . . . . . . . . . . . . . Rochester
Mynter, Herman, M. D. . . . . . . . . . . . . . . . . . . . . . . Buffalo
Packard, John H., M. D. . . . . . . . . . . . . . . . . . . . . . Philadelphia
Parmenter, John, M. D. . . . . . . . . . . . . . . . . . . . . . . Buffalo
Park, Roswell, M. D. . . . . . . . . . . . . . . . . . . . . . . . Buffalo
Penrose, Charles B., M. D. . . . . . . . . . . . . . . . . . . . Philadelphia
Potter, E. B., M. D. . . . . . . . . . . . . . . . . . . . . . . . . Rochester
Potter, Frank Hamilton, M. D. . . . . . . . . . . . . . . . . . . . Buffalo
Potter, William Warren, M. D. . . . . . . . . . . . . . . . . . . . Buffalo
Price, Joseph, M. D. . . . . . . . . . . . . . . . . . . . . . . . Philadelphia
Putnam, James Wright, M. D. . . . . . . . . . . . . . . . . . . . . Buffalo
Renner, W. Scott M. D. . . . . . . . . . . . . . . . . . . . . . . Buffalo
Richmond, Nelson G., M. D. . . . . . . . . . . . . . . . . . . . . Fredonia
Rochester, De Lancey, M. D. . . . . . . . . . . . . . . . . . . . . Buffalo
Roe, John O., M. D. . . . . . . . . . . . . . . . . . . . . . . . . Rochester
Schultze, Bernhard Sigmund, M. D. . . . . . . . . . . . . . . . . . Jena
Sellstedt, Lars Gustav, Esq., . . . . . . . . . . . . . . . . . . . Buffalo
Smith, Eugene A., M. D. . . . . . . . . . . . . . . . . . . . . . . Buffalo
Tait, Lawson, Esq., M. D. . . . . . . . . . . . . . . . . . . . . Birmingham
Vander Veer, A., M. D. . . . . . . . . . . . . . . . . . . . . . . . Albany
Weeks, H. M., M. D. . . . . . . . . . . . . . . . . . . . . . . . . Trenton
Weigel, Louis A., M. D. . . . . . . . . . . . . . . . . . . . . . . Rochester
Wende, Ernest, M. D. . . . . . . . . . . . . . . . . . . . . . . . . Buffalo

### LIST OF ILLUSTRATIONS.

PAGE.

AN ANTISEPTIC LIGATURE BOX (H. M. WEEKS).
Fig. 1.—Showing box closed . . . . . . . . . . . . . . . . . . . . . . 117
Fig. 2.—Showing box open . . . . . . . . . . . . . . . . . . . . . . . 118

REMINISCENCES OF FIELD HOSPITAL SERVICE WITH THE ARMY OF THE POTOMAC (W. W. POTTER).
Plate I.—Regimental Hospital 49th N. Y. Vols., facing . . . . . . . . . 137
Plate II.—Field Hospital Savage's Station, Va., facing . . . . . . . . . 139
Plate III.—First Division Hospital Second Army Corps, facing . . . . } 193
Second Division Hospital Second Army Corps, facing . . . . } 193
Plate IV.—Third Division Hospital Second Army Corps, facing . . . . . 199

AN EASY METHOD OF REPAIRING THE PERINEUM (J. C. DA COSTA).
Fig. 1.—Bar one-half size . . . . . . . . . . . . . . . . . . . . . . . 230
Fig. 2.—Surface denuded and stitches in place . . . . . . . . . . . . . 230
Fig. 3.—Operation finished and bars in place . . . . . . . . . . . . . 231

RECTIFICATION OF FACE PRESENTATIONS (ROLLIN L. BANTA).
Figs. 1–3.—Diagrams of inlet and outlet of pilvis . . . . . . . . . . . 258
Fig. 4.—Diagram showing sacro-cotyloid diameters, and sacro-cotyloid spaces . . . . . . . . . . . . . . . . . . . . . . . . . . . . . . 259

DERMOID CYST OF LEFT OVARY (W. W. POTTER), WITH PATHOLOGICAL DEMONSTRATION (W. C. KRAUSS).
Fig. I.—Showing interior of cyst with development of teeth . . . . . . . 273

HEMORRHOIDS: THEIR DIAGNOSIS AND TREATMENT (EDWD. CLARK).
Fig. 1.—Daggett Examining Table, arranged for dorsal position . . . . . 408
Fig. 2.—Daggett Examining Table, arranged for recumbent position . . . 408
Fig. 3.—The Author's Antiseptic Rectal Tampon . . . . . . . . . . . . 414

AN IMPROVED TAPE MEASURE (W. C. KRAUSS).
Fig. . . . . . . . . . . . . . . . . . . . . . . . . . . . . . . . . . . 576

A SYMPOSIUM OF ABDOMINAL SURGERY (L. S. MCMURTRY, JOSEPH PRICE, CHAS. B. PENROSE, AND JOHN H. PACKARD).
Plate.—Residence and office of Dr. Ephraim McDowell where the first ovariotomy was performed, facing . . . . . . . . . . . . . . . . . . 593

### INDEX.

### A.

PAGE.
Abdominal Surgery, Symposium of . . 559
Abdominal Dropsy in a Young Subject. By James Wright Putnam, M. D. . . . . . . . . . . . . . . . 277
Abdominal Sections, A Report of One Hundred and Sixty, with Special Reference to the Prevention of Unpleasant Sequelæ. Matthew D. Mann, A. M., M. D. . . . . . . . . 535
Academy of Medicine, New York, 469, 679
Academy of Sciences, Paris. Translation. By A. Dagenais, M. D. . . . 220
Accidents and Complications Incident and Subsequent to Abdominal and Pelvic Operations. By Joseph Hoffman, M. D. . . . . . . . . . . . . 206
Adenoid Vegetations of the Naso-Pharynx. W. Scott Renner, M. D., C. M. . . . . . . . . . . . . . . 523
American Medical Association . . . . 764
. . . . . . . . 44, 392, 499, 584, 695
American Society of Microscopists,
. . . . . . . . . . . 50, 124, 703, 767
American Association of Obstetricians and Gynecologists 51, 124, 168, 179,
. . . . . . . . . . . . . . . 245, 704
American Pediatric Society . . . . . 123
American Gynecological Society . . . 58
. . . . . . . . . . . . . . . . 123, 179
American Physicians, Association of . 123
American Public Health Association . 178
American Academy of Medicine . . . 256
Anatomical Nomenclature, Reform in . 698
Anecdote Concerning Dr. Ricord . . 488
Animal Alkaloids . . . . . . . . . . 589
Animal Physiology, A Text-Book of . 319
Anatomy, An Introduction to Pathology and Morbid . . . . . . . . 251
Antiseptic Ligature Box. By. H. M. Weeks, M. D. . . . . . . . . . 117
Annual of the Universal Medical Sciences . . . . . . . . . . . . . . 187
Annals of Surgery . . . . . . . . . 391
Application and Advantages of Celluloid-Ring Pessaries, as made by the Rhenish Rubber and Celluloid Factory, Mannheim. By Dr. Bernhard Sigmund Schultze . . . . . . . . 364
Army of the Potomac, etc. By W. W. Potter, M. D. . . . . . . . . . 137, 193
Asthma Reflex, especially of Nasal Origin. Frank Hamilton Potter, M. D. . . . . . . . . . . . . . . 463
PAGE.
Ayres, C. S., A. M., M. D. . . . . . 498

### B.

Bacteria and Their Relation to Health and Disease, The Story of . . . . 326
Banta, Rollin L., M. D. . . . . . . . 177
Banta, Rollin L., M. D. Rectification of Face Presentations . . . . . . 257
Belford's Magazine . . . . . . . . . 456
Belfield, Dr. Wm. T. . . . . . . . . . 712
Belladona Poisoning — Profuse Epitaxis — Recovery. William C. Krauss, M. D. . . . . . . . . . . 483
Bergstrand, Dr. A. Case of Inversio Uteri Totalis Post Partum — Acute Anemia — Intravenous Transfusion of Common Salt Solution — Recovery . . . . . . . . . . . . . . . 161
Best Serial Stories . . . . . . . . . 256
Billings, Frank S., M. D. . . . . . . . 244
Billings, Frank S. The Cornstalk Disease in Cattle . . . . . . . . . 1, 65
Books and Pamphlets Received . . . 62, 133, 189, 254, 454, 510, 590, 654, 706,
. . . . . . . . . . . . . . . . . . 776
Book on the Physician Himself and Things that Concern His Reputation and Success . . . . . . . . . . . . 132
Bradnack, F., M. D. Death: Its Modes, Signs, and Premonitions, 667, 718
Bright's Disease, Lectures on . . . . 61
Bromidia . . . . . . . . . . . . . . 135
Brown-Séquard Fluide Testiculaire . 119
Brooks, A. J., M. D., Obituary . . . 735
Buffalo Medical and Surgical Association . . 25, 91, 289, 366, 541, 626, 689
Buffalo Pathological Society . . . . 99, 234, 292, 326, 383, 387, 417, 543, 688
Buffalo Street-Car Nuisance Again . . 443
Buffalo Obstetrical Society . 478, 568, 686
. . . . . . . . . . . . . . . . . 737

### C.

Calf Pepsin . . . . . . . . . . . . 779
Canadian Medical Association . . . . 179
Canvasser, The Professional . . . . . 64
Carcinoma of the Pancreas . . . . . 728
Cary, Charles, M. D. . . . . . . . . . 177
Case of Inversio Uteri Totalis Post Partum — Acute Anemia — Intravenous Transfusion of Common Salts Solution — Recovery. By Dr. A. Bergstrand . . . . . . . . 161

PAGE.
Case of Persistent Facial Neuralgia, of Reflex Dental Origin . . . . . . 487
Cash, Merritt H., Prize Essay . . . . 577
Cattle Disease in Nebraska . . . . . 323
Change of Life, She Thought it Was Her . . . . . . . . . . . . . . . 713
Chemistry : Text-Book of Medical . . 505
Chemistry : General, Medical, and Pharmaceutical . . . . . . . . . 385
Chicago Policlinic . . . . . . . . . 651
Christian Science, Check to . . . . . 712
Clark, Edward, M. D. . . . . . . . . 53
Clark, Edward, M. D. Hemorrhoids, Their Diagnosis and Treatment . . 406
Clark, Charles P., M. D. . . . . . . 245
Cocaine in Surgery, The Status of . . 305
Codeine for Morphine Habit . . . . 171
Congenital Umbilical Hernia. By John Parmenter, M. D. . . . . . 347
Congress of American Physicians and Surgeons . . . . . . . . . . . . 246
Constipation : Causes, Consequences and Rational Treatment of. By De Lancey Rochester, M. D. . . . . 214
Colon, Treatment of Lesions of. . . . 658
College of Pharmacy . . . . . . . . 124
Cornstalk Disease in Cattle. Frank S. Billings . . . . . . . . . . . 1, 65
Committee on Hygiene . . . . . . . 243
Coskery, Oscar J., M. D. . . . . . . 52
Cosmopolitan, The . . . . 389, 512, 591
Crego, F. S., M. D., Foreign Letters . . . . . . . . . . . . . . . .591, 642
Crego, Dr. Floyd S. . . . . 498, 586, 701
Cremation of the Dead . . . . . . . 328
Criticism of Some Recent Utterances on Ectopic Gestation. By Lawson Tait, F. R. C. S. E. . . . . . . . . 329
Cushing, Clinton, M. D. Ligatures and Sutures — What Materials Shall be Used ? . . . . . . . . . . . . 340
Cushing Clinton, M. D. . . . . . . 586
Cyclopedia of the Diseases of Children . . . . . . . . . . . . 53, 246

### D.

Dagenais, Dr. A., Gleanings from Foreign Journals . . . . . . . . . 164
Daniels, Dr. C. M. . . . . . . . . 498
Davis, Treatment of Peritonitis . . . 750
Death, Its Modes, Signs and Premonitions, F. Bradnack, M. D. . . . . 667
Dermoid Cyst of the Left Ovary — Operation, Recovery. By Wm. Warren Potter, M. D., and Wm. C. Krauss, M. D. . . . . . . . . . . 272
Detroit Free Press Souvenir. . . . . 456
Devening, Daniel, M. D., Obituary . 736
Diabetes Mellitus and Insipidus . . . 509
Diagnostic Tampon of Schultze . . . 174
PAGE.
Diagnosis and Treatment of Functional Disorders of the Stomach, by Frederick P. Henry, M. D. . . . . 263
Dimon, Theodore, M. D. . . . . . . 125
Diseases of Women, Massey's Electricity in the . . . . . . . . . . . 37
Diseases of the Nose and Throat, A Treatise on. By Francke Huntington Bosworth, A. M. M. D. . . . . 449
Disordered Digestion and Dyspepsia . 189
Dixie Doctor, The . . . . . . . . . 444
Dixon-Jones Case, The. . . . . . . 580
Dorland, E. T., M. D. . . . . . . . . 245
Drainage in Abdominal and Pelvic Surgery . . . . . . . . . . . . 605

### E.

Ectopic Gestation, etc. Tait . . . . 329
Education and Culture as relating to the Health and Diseases of Women. 509
Edgren, Prof. T. G. Experiments with Calomel as a Diuretic . . . . . . 156
Effects of Mental Impressions . . . 489
Eighty-Fourth Annual Meeting of the Medical Society of the State of New York. . . . . . . . . . . . . . 383
Electricity in Gynecological Practice . 626
Eliot, Llewellyn, M. D. Rare Sequelæ of Typhoid Fever. . . . . . . 598
Emmet, Bache McE., M. D. . . . . 318
Epitome of Examination of Recruits . 711
Errors of Refraction . . . . . . . . 183
Essentials of Pathology and Morbid Anatomy. . . . . . . . . . . . 186
Essentials of Materia Medica . . . . 386
Etiology of Recurring Facial Neuralgia after Neurectomy of one of the Affected Branches of the Fifth Nerve . . . . . . . . . . . . . 370
European Journals, Gleanings from. By W. C. K. . . . . . . . . . . 89
Excerpts. John A. Miller, Ph. D. . . . . . . . . . . . . 309, 640, 689, 745
Exhibition, The Paris . . . . . . . 328
Experiments with Calomel as a Diuretic. By Prof. J. G. Edgren . . . 156
Experiments on the Proliferation and Development of Leucocytes . . . . 484
Eye, Text-Book on Diseases of. . . 702

### F.

Face, Presentations, Rectification of. . 257
Faculty Change — Niagara University. 317
Faculty of the College of Physicians and Surgeons of Baltimore . . . . 51
Fecundity, Remarkable . . . . . . . 749
Festal Number of Hygiea . . . . . . 375

PAGE.

Fetus Papyraceus, A Case of. By E. W. Mulligan, M. D. . . . . . . 24
Foreign Letters: Crego . . . . . 591, 642

### G.

Gaillard's Medical Journal . . . . . 444
Gardner, Edwin F., Asst. Surgeon, U. S. A. . . . . . . . . . . . . . . 318
Gastric and Duodenal Ulcers. Clarence King, M. D. . . . . . . . . . 457
Gastro-Intestinal Diseases of Pregnancy . . . . . . . . . . . . . . 568
Germania. . . . . . . . . . . . . . 385
Gleanings from French Journals, by Dr. A. Dagenais. . . . . . . . . 164
Gleanings from European Journals. By W. C. K. . . . . . . . . . . . 89
Goitre, Congenital Exopthalmic . . . 750
Goldberg, Jacob, M. D. . . . . . . 318
Grant, H. Y., M. D. . . . . . . . 177
Granger, Wm. D., M. D. . . . . . 701
Great Thing, The Beginnings of a . 759
Gregory, W. G., M. D. . . . . . . . 53
Greene, W. D., M. D. . . . . . . . 447
Greene, Joseph C., M. D. . . . . . 245
Grosvenor, J. W., M. D., Therapeutic Value of Alcohol . . . . . . . . 347
Guide to Diseases of Children . . . 589

### H.

Hanks, Horace F., M. D. . . . . . 318
Hand-Book of Pathological Anatomy and Histology. . . . . . . . . . 502
Hand-Book of Materia Medica, Pharmacy and Therapeutics . . . . . 705
Harderfold Fabric Co. . . . . . . . 779
Harvey, Thos. B., M. D. . . . . . . 384
Hayd, H. E., M. D., Electricity in Gynecological Practice . . . . . . 626
Health Law in Michigan, A New . . 242
Health Physician, The . . . . . . . 315
Henry, Frederick P. M. D., Diagnosis and Treatment of Functional Disorders of the Stomach . . . . . 263
Hemorrhoids — Their Diagnosis and Treatment. By Edward Clark, M. D. 406
Heredity of Tuberculosis . . . . . 170
Highway Improvements . . . . . . 315
Hoffman, Joseph, M. D., Accidents and Complications Incident and Consequent to Abdominal and Pelvic Operations . . . . . . . . . . . 206
Hoffman, Joseph, M. D., the Treatment of Lesions at and About the Head of the Colon . . . . . . . 658
Holiday Number of the Trained Nurse. 456
Holmes, Oliver Wendell's Birthday . 136

PAGE.

How Much Should a City Pay its Health Officer? . . . . . . . . . 244
Howe, Dr. L. . . . . . . . . . . . 446
Howell, S. Y., M. D., Pathogenesis and Morbid Anatomy of Tumor Albus . . . . . . . . . . . . . . 543
Hubbell, Alvin A., Optic Neuritis and its Significance as a Symptom . . . 398
Hunter, James B., M. D. . . . . . . 52
Hygienic Underwear . . . . . . . . 729
Hysteria . . . . . . . . . . . . . . 153
Hygeia, Festal Number of . . . . . 375
Hyoscine Hydrobromate as a Hypnotic. By E. B. Potter, M. D. . . . . 86
Hypnotism — Its History and Present Development. By Frederick Björnström, M. D. . . . . . . . . . . 453
Hysteria. By James Wright Putnam, M. D. . . . . . . . . . . . . . . 153

### I.

Immunity through Leucomaines . . 56
Improved Tape Measure. Wm. C. Krauss, M. D. . . . . . . . . . . 576
Index Catalogue of the Library of the Surgeon General's Office, U. S. A. . 386
Inebriety—Its Etiology, Pathology, Treatment and Jurisprudence . . . 188
Infants' Summer Hospital . . . . . 123
Influence of Oral Irritation in the production of Diseases of the Upper Air Tract. By Frank Hamilton Potter, M. D. . . . . . . . . . . . 12
Interleaving of Advertising Pages between Reading Matter . . . . . . 382
International Congress for Study of Alcoholism . . . . . . . . . . . 116
International Congress . . . . . . . 123
International Medical Congress, The., 583
International Medical Annual . . . 653
Intermittent Fever and Atmospheric Temperture . . . . . . . . . . . 240
Internal Œsophagotomy. John O. Roe, M. D. . . . . . . . . . . . 593
Intestine, Treatment of Gangrene of . 607
Intestinal Anastomosis with Segmented Rubber Rings . . . . . . 313
Introduction to Pathology and Morbid Anatomy . . . . . . . . . . . . . 251
Intubation of the Larynx . . . . . . 710
Instantaneous Cure of Whooping Cough . . . . . . . . . . . 173, 237
Inversio Uteri Totalis Post Partum, Acute Anemia, Intravenous Transfusion of Common Salt Solution, Recovery. By Dr. A. Bergstrand . 161
Iodoform . . . . . . . . . . . . . 175
Iowa State Medical Society . . . 560
Ivory Gate, Through the . . . . . . 580

PAGE.

### J.

Jackson, Dr. J. D. . . . . . . . . 711
Jefferson, Thos., and the University of Virginia . . . . . . . . . . . 129
Jessel, Henry K. . . . . . . . . . . 317
Jenks, the William F. Memorial Prize . . . . . . . . . . . . . . . 709
Johns Hopkins' Hospital . . . . . . 327
Jones Case, The Dixon . . . . . . . 580

### K.

Kibler, Dr. C. B. . . . . . . . . . . 585
Kilmer's Physician's Pocket Ledger . 192
King, A. F. A., M. D. . . . . . . . . 693
King, Thomas J., M. D. . . . . . . . 318
King, Clarence, M. D., Gastric and Duodenal Ulcers . . . . . . . . 457
Krauss, Wm. C., M. D., with Wm. Warren Potter, M. D., Dermoid Cyst of the left Ovary, Operation, Recovery . . . . . . . . . . . 272
Krauss, Wm. C., M. D., Acute Belladonna Poisoning, Profuse Epistaxis, Recovery, 483. An Improved Tape Measure . . . . . . . . . . . . . 576
Krauss, Wm. C., M. D. . . 125, 177, 651

### L.

Laboratory Guide in Urinalysis and Toxicology . . . . . . . . . . . 133
Labor Pains and After Pains . . . . 737
Lanoline Ointments, Some New. Dr. Ernest Wende . . . . . . . . . . 20
Lassar's Method for Treating the Scalp. Wende . . . . . . . . . . 441
Laparatomy, Six Cases of, Packard . . 613
Lectures on Bright's Disease . . . . 61
Lectures on Obstetric Nursing . . . 253
Lehrbuch der Hebammenkunst . . . 588
L'Enseignement et L'Organization de L'Art Dentaire aux E'tats unis . . 186
Lewis, Daniel, M. D. . . . . . 446, 701
Ligatures and Sutures—What Materials Shall be Used? By Clinton Cushing, M. D. . . . . . . . . . 340
Lippincott, J. B. Co., Regional Anatomy . . . . . . . . . . . . . . 780
Literary Notes . . . . . . 64, 136, 191, 256, 591, 327, 391, 456, 511, 709
Lunatic Asylum, The New York State . 444
Lunacy Legislation, New . . . . . . 649

### M.

Mallon, Mrs. Isabel . . . . . . . . 770
Manton, W. P., M. D. . . . . . . . . 244
Manual of Chemistry, for the Use of Medical Students . . . . . . . . 387
Manual of Obstetrics . . . . . . . . 325

PAGE.

Manual of Organic Materia Medica. 653
Mann, Matthew D., A. M. M. D., A Report of 160 Abdominal Sections, with Special Reference to the Prevention of Unpleasant Sequelæ . . 535
Mastin, C. H., M. D. . . . . . . . . . 246
Massey's Electricity in the Diseases of Women . . . . . . . . . . . . 37
Maus, Louis M., Asst. Surgeon, U. S. A. . . . . . . . . . . . . . . . 318
Maryland Medical Journal. . . . . . 711
McDowell Medical Society . . . . . 246
McMillan, Dr. Charles . . . . . . 445
McMurtry, Dr. L. S. . . . . . . 444, 498
McMurtry, L. S., M. D., Operative Methods Illustrated by the History of Ovariotomy in America . . . . 599
McPherson, Dr. G. W. . . . . . . 446
Medical and Surgical Reporter . . . 390
Medical Association, State of Alabama. . . . . . . . . . . . . . 585
Medical Mirror . . . . . . . . . . . 328
Medical Association of Central N. Y. . . . . . . . . . . . . . . . 317, 701
Medical Society of Erie County . 383, 733
Medical Society of the State of New York . . . . . . . 245, 383, 447, 493
Medical Department of the Niagara University . . . . . . . 180, 582, 647
Medical Department of the University of Buffalo . . . . . . . . . . 179, 578
Medical College Notes . . . . . . . 768
Medical Examiners Law, The New . 751
Medical Professions of the States of New York, Ohio, Illinois, Indiana and Iowa. . . . . . . . . . . . . 710
Medico-Chirurgical College of Philadelphia. . . . . . . . . . . . . 52
Microscope in the Diagnosis of Disease, The Importance of. By E. W. Mulligan, M. D. . . . . . . . 148
Microscope, The. . . . . . . . . . 256
Miller, John A., M. D. . . . . . . 179, 309
Mink, Arthur E., M. D., Pathogenesis of Epilepsy. . . . . . . . . . . . 17
Mississippi Valley Medical Assoc'n . 123
Miller, John A., Ph. D., Excerpts by . 745
Mirror, The Medical . . . . . . . . 328
Mississippi Valley Medical Association . . . . . . . . . . . . . . 768
Missouri Medical College, The . . . 651
Monthly Nursing . . . . . . . . . 588
Mortality and Vital Statistics for the Eleventh Census . . . . . . . . . 122
Mott, Alexander B., M. D. . . . . . 125
Mulligan, E. W., M. D., A Case of Fetus Papyraceus, 24—The Importance of the Microscope in the Diagnosis of Diseases. . . . . . . 148
Mynter, Herman, M. D., Perityphlitis and Its Surgical Treatment . . 513

PAGE.

### N.

National Medical Dictionary. . . . . 504
National Magazine, The . . . . . . 391
National Association of Railway Surgeons . . . . . . . . . . . . . . . 585
Nervo-Vascular System. . . . . . 254
Nervous Reflexes of Nasal Origin . . 486
Neuropathic Origin of Edema . . . . 312
Neuralgia, Etiology of Recurring Facial . . . . . . . . . . . . . . 370
Neuralgia, Insufflation of Salts in the Treatment of . . . . . . . . . . 489
Neuralgla, Case of Persistent Facial, of Dental Origin . . . . . . . . . . 487
New Journal, Medical Mirror . . . . 328
New York State Lunatic Asylum . . 444
New York Academy of Medicine. Sect. on Orthopedic Surgery. . 469, 679
New York Physicians Mutual Aid Association . . . . . . . . . . . 511
New Lunacy Legislation . . . . . 649
North Carolina Medical Journal . . 456
North of England Obstetrical and Gynecological Society . . . . . . 499
Noses, Deformed, Cure of . . . . . 590
Noyes: Text-Book on Diseases of the Eye . . . . . . . . . . . . 702
Nursing, Obstetric, Lectures on . . . 253

### O.

Obstetrics, Text-Book of . . . . . . 587
Obstetrical Society of Philadelphia . . . . . . . . . . . . . . . 278, 229, 431
Obstetric Nursing, Lectures on . . . 253
Œsophagotomy, Internal. J. O. Roe . 593
Oliver Wendell Holmes' Birthday . . 136
Ophthalmology and Ophthalmoscopy for Practitioners and Students of Medicine . . . . . . . . . . . . . 249
Optic Neuritis and Its Significance as a Symptom. By Alvin A. Hubbell, M. D. . . . . . . . . . . . . . . 398
Oral Irritation in the Production of Disease of the Upper Air Tract. By Frank Hamilton Potter, M. D. . 12
Original Investigation of the Cattle Disease in Nebraska . . . . . . . 323
Ourselves as Others See Us . . . . 316
Ovary, Dermoid Cyst of . . . . . . 272
Ovariotomy in America. By McMurtry . . . . . . . . . . . . . . . . . 599

### P.

Packard, John H., M. D., Six Cases of Laparatomy . . . . . . . . . 613
Park, Dr. Roswell . . . . . . . . 585
Park, Roswell, M. D., Surgery of the Alimentary Canal . . . . . . . 634

PAGE.

Park, Davis & Co.'s Seasonable Suggestions . . . . . . . . . . . . . 780
Paris Exhibition . . . . . . . . . 328
Parmenter, John, M. D., Congenital Umbilical Hernia . . . . . . . . . 347
Parmenter, John, M. D., Treatment of Tumor Albus . . . . . . . . . . 557
Patents, Recent Medical . . . . . . 777
Pathogenesis of Epilepsy. By Arthur E. Mink, M. D. . . . . . . . . . . 17
Pasteur Institute, The . . . . . . . 485
Penrose, C. B., Gangrene of Intestine . . . . . . . . . . . . . . 607
Peritonitis, Treatment of, by W. E. B. Davis, M. D. . . . . . . . . . . 750
Perityphlitis and Its Surgical Treatment. Herman Mynter, M. D. . . 513
Philadelphia County Medical Society, . . . . . . . . . . . . . . 221, 599
Philadelphia Medical and Surgical Reporter . . . . . . . . . . . . 390
Phimosis, Reflex Symptoms Due to . . 169
Photographic Illustrations of Skin Diseases . . . . . . . . . . . . . 253
Physiology of the Domestic Animals . 131
Physicians' Leisure Library . . . . . 60
Physician and His Journals . . . . . 172
Physicians' Mutual Aid Association, New York . . . . . . . . . . . . 511
Physiology, Text-Book of. . . . . . 508
Physiological Notes on Primary Education and the Study of Language . 61
Physiological Action of Thallin, On the . . . . . . . . . . . . . . . 372
Physicians' Hand-Book for 1890. By A. D. Elmer, M. D. . . . . . . . 453
Potter, Wm. Warren, M. D. . . . . 53
Potter, William Warren, M. D. Reminiscences of Field Hospital Service with the Army of the Potomac, 137, 193
Potter, Wm. Warren, M. D., with Wm. C. Krauss, M. D., Dermoid Cyst of the Left Ovary, Operation, Recovery . . . . . . . . . . . 272
Potter, E. B., M. D., Hyoscine Hydrobromate as a Hypnotic . . . . . 86
Potter, Frank Hamilton, M. D., Influence of Oral Irritation in the Production of Diseases of the Upper Air Tract . . . . . . . . . . . 12
Potter, Frank Hamilton, M. D., Reflex Asthma, especially of Nasal Origin, 463
Practical Electricity in Medicine and Surgery . . . . . . . . . . 136, 703
Pregnancy, Gastro-Intestinal Diseases of . . . . . . . . . . . . . . . 568
Preston Retreat, The . . . . . . . 48
Prescription for Psoriasis . . . . . . 488
Progress of Inventions Since 1845 . . 191
Price, Joseph, M. D. . . . . . . . 125

PAGE.

Price, Joseph, M. D., Drainage in Abdominal and Pelvic Surgery . . 605
Prince, Dr. David . . . . . . . . . 445
Private Hospital for Rectal Diseases . 64
Proceedings of the American Society of Microscopists . . . . . . . . . 703
Proposed Reform in Medical Education . . . . . . . . . . . . . . . . 581
Protection Applied to the Practice of Medicine . . . . . . . . . . . . 380
Psychoses, The Treatment of, by Opium 163
Pus. By Roswell Park, M. D. . . . 292
Putnam, James Wright, M. D., Abdominal Dropsy in a Young Subject. 277

### Q.

Quinine, Neat Way of Giving . . . . 751

### R.

Railway Surgeons, National Association of . . . . . . . . . . . . . . 585
Rapid Transit for Physicians . . . . 496
Recent Medico-Legal Decisions . 165, 238
Rectal Diseases, The Private Hospital for . . . . . . . . . . . . . . . 64
Rectification of Face Presentations. By Rollin L. Banta, M. D. . . . . . 257
Reference Hand-Book of the Medical Sciences . . . . . . . . . . . . . 324
Reflex Symptoms Due to Phimosis . . 169
Regional Anatomy . . . . . . . . . 780
Relation of the Thoracic and Abdominal Walls to the Spinal Column, Considered with Reference to the Treatment of Antero-Posterior Curvatures. By Louis A. Weigel, M. D. . . . . . . . . . . . . . . . 393
Reminiscences of Field Hospital Service with the Army of the Potomac. By William Warren Potter, M. D. . . . . . . . . . . . . . 137, 193
Renner, Dr. W. Scott . . . . . . . 701
Renner W. Scott, M. D. C. M., Adenoid Vegetations of the Naso-Pharynx . . . . . . . . . . . . 523
Report of Stevens' Commission . . . 495
Reprints, Uniformity in . . . . . . . 173
Richmond, N. G., M. D., Carcinoma of the Pancreas . . . . . . . . . 728
Ricord, Professor . . . . . . . . . 246
Roberts, John B., M. D. . . . . . . 177
Rochester, DeLancey, M. D., Gastro-Intestinal Diseases of Pregnancy . 568
Rochester Infants' Summer Hospital . 123
Rochester, Mrs. Margaret Munro . . 445
Rochester, DeLancey, M. D., Causes, Consequences and Rational Treatment of Constipation . . . . . . . 214
Roe, John O., M. D., Internal Œsophagotomy . . . . . . . . . . . . . 593

PAGE.

Rohé, Dr. Geo. H. . . . . . . . . . 701
Romanes, Geo. J. . . . . . . . . . . 64
Ross, Dr. T. Haven . . . . . . . . 701

### S.

Sayre, Lewis Hall, M. D. . . . . . 446
Scientific American . . . . . . . . . 191
Science and Practice of Midwifery, Treatise on . . . . . . . . . . . . 252
Schultze, Dr. Bernhard Sigmund, Application and Advantages of Celluloid Ring Pessaries . . . . . . . . 364
"She Thought it was Her Change of Life" . . . . . . . . . . . . . . . 713
Sick Benefit, Funeral Aid, Death Beneficiary Associations . . . . . . 712
Sixteenth Annual Report of the State Commissioner in Lunacy for the Year 1888 . . . . . . . . . . . . 62
Skin Diseases, Photographic Illustrations of . . . . . . . . . . .
Smith, E. A., M. D., Symptomatology and Diagnosis of Tumor Albus . . 551
Smith, J. Lewis, M. D. . . . . . . . 712
Southern Surgical and Gynecological Association . . . . . . 178, 246, 316
Society Transactions, Review of . . . 58
Spinal Concussion . . . . . . . . . . 651
Spine, Antero-Posterior Curvatures of. By L. A. Weigel, M. D. . . . . . 393
Squire, Dr. Truman Hoffman . . . . 445
Statistical Report of Operations Performed in Two Years at the Saint Louis . . . . . . . . . . . . . . 373
Statistics of Leprosy in the U. S. . . 392
Status of Cocaine in Surgery . . . . 305
State Licensing Authority . . . . . . 763
Stockton, Chas. G., M. D. . . . 177, 585
Stomach, Functional Diseases of . . . 263
Story of Bacteria and Their Relations to Health and Disease . . . . . . 326
Stevens Commission, Report of . . . 495
Street Car Nuisance Again . . . . . 443
Street Car and Public Health . . . . 46
Strong, Dr. Phineas H. . . . . . . . 497
St. Louis Polyclinic . . . . . . . . 444
Surgical Hand-Book for the Use of Practitioners and Students . . . . 325
Surgery of the Alimentary Canal . . 634
Surgery, Principles and Practice of . . 770
Suspension, the Present Status of in the Treatment of Locomotor Ataxia. 692
Sutures and Ligatures, Cushing . . . 340
Swedish Movement and Massage Treatment . . . . . . . . . . . . 57
Syllabus of the Obstetrical Lectures in the Medical Dept. of the University of Pennsylvania . . . . . . . . . . 652
Symposim of Abdominal Surgery . . 599
Synopsis of Human Anatomy . . . . 59

PAGE.

System of Obstetrics by American Authors. By Hirst . . . . . . . 127
Syphilis of the Nervous System . . . 189

T.

Tait, Lawson, F. R. C. S. E., Criticism of Some Recent Utterances on Ectopic Gestation . . . . . . . . 329
Tape Measure, Improved . . . . . . 576
Tarrants Seltzer Aperient . . . . . . 780
Taylor, Isaac E., M. D. . . . . . . . 318
Tenth International Medical Congress 178
Tennessee State Medical Society . . 585
Text-Book of Medical Chemistry . . 505
Text-Book of Human Physiology . . 508
Text-Book of Obstetrics . . . . . . 587
Text-Book on Diseases of the Eye . . 702
Therapeutic Value of Alcohol. By J. W. Grosvenor, M. D. . . . . . . . 347
Through the Ivory Gate . . . . . . 500
Thornton, Wm. H., M. D., Labor Pains and After Pains . . . . . . . . . 737
Trained Nurse, Holiday Number of the . . . . . . . . . . . . . . . 456
Transatlantic, The. . . . . . . 256, 390
Transactions of the American Surgical Association . . . . . . . . . . . 180
Transactions of the American Association of Obstetricians and Gynecologists . . . . . . . . . . 328, 391, 704
Treatment of Lesions at and About the Head of the Colon, Joseph Hoffman, M. D. . . . . . . . . . 658
Treatise on Materia Medica, Pharmacology, and Therapeutics . . . . . 507
Treatment of Dysmenorrhea by Hypodermic Injections of Phenic Acid . . . . . . . . . . . . . . 489
Treatment of Syncope . . . . . . . 488
Treatment of Psychoses by Opium . . 136
Treatment of Hemorrhoids by Chysarobine . . . . . . . . . . . 487
Tremaine, Wm. S., Surgeon, U. S. A. . . . . . . . . . . . . . . . . 177, 318
Treatise on Diseases of the Nose and Throat. By Francke Huntington Bosworth, M. D. . . . . . . . . . 449
Treatise on the Science and Practice of Midwifery . . . . . . . . . . 252
Tri-State Medical Association . . . 177
Tumor Albus . . . . . . . . . . . 543
Typhoid Fever, Rare Sequelæ of, Llewellyn Eliot, M. D., . . . . . 598

U.

Umbilical Hernia, Congenital . . . . 347
Uniformity in Reprints . . . . . . 173
University of Buffalo, Medical Department . . . . . . . . . . . . . . 576

PAGE.

University of Niagara, Medical Department . . . . . . . . . . . . . . 647
Urine, The Common Poisons, and the Milk . . . . . . . . . . . . . 385
Uteri, Inversio Totalis . . . . . . . 161

V.

Valuable and Unique Business Calender . . . . . . . . . . . . . . . 389
Vander Veer, A. M. D., "She Thought it was Her Change of Life." . . . . . . . . . . . . . . 713
Volkmann, Richard Von, Prof. . . . 384
Volume XXIX. . . . . . . . . . 46, 756

W.

Warner, Wm. R. & Co. . . . . . . . 136
Weigel, Louis A., M. D. Relation of the Thoracic and Abdominal Walls to the Spinal Column, Considered with Reference to the Treatment of Antero-Posterior Curvatures . . . . . . . . . . . . . . 393
Wende, Ernest, M. D., Lassar's Treatment of the Scalp — Translation . . . . . . . . . . . . . .
Wende, Ernest, M. D., Some New Lanoline Ointments — Translation. 20
Weeks, H. M., M. D., Antiseptic Ligature Box . . . . . . . . . . . . 117
Western Pennsylvania Medical College, The Fourth Annual Commencement . . . . . . . . . . . 651
Widner, Joe Elmer, M. D. . . . . . 498
Wilson, Ellwood, M. D. . . . . . . . 52
Winckel: Text-Book of Obstetrics . 587
Women, Sanitary Organization of . . 763
Women, How They Should Sit . . . 779
Wood's Medical and Surgical Monographs . 59, 133, 188, 250, 322, 506, 586
Wood, Dr. Charles S. . . . . . . . . 498
Worthington & Co., Messrs. . . . . 392
Wyeth, John A. and Bro. . . . . . . 135

Y.

York, Dr. G. W. . . . . . . . . . . 498

### Editorials :

American Medical Association . . . 44
Echoes from the Nashville Meeting . . . . . . . . . 764
Nashville Meeting . . . . 695
Association of Obstetricians and Gynecologists . . . . 168
Associations National Specialists' . . . 762
Beginnings of a Great Thing . . . . 759

PAGE.

Board of Medical Examiners, State of New York . . . . . . . . . . . . . 758
Brown-Séquard Fluide Testiculaire . 119
Buffalo Street Car Nuisance Again . . 443
Catalogue of Society Transactions . . 762
Codeine for Morphine Habit . . . . 171
Committee on Hygiene . . . . . . 243
Diagnostic Tampon of Schultze . . 175
Dixon Jones Case . . . . . . . . . . 580
Festal Number of *Hygiea* . . . . . . 375
Forty-fourth Annual Commencement of the Medical Department, University of Buffalo . . . . . . . . . . 578
Gaillard's Medical Journal . . . . . 444
Health Physician . . . . . . . . . . 315
Heredity of Tuberculosis . . . . . 169
Highway Improvements . . . . . . 315
How Much Should a City Pay Its Health Officer? . . . . . . . . . . 244
Interleaving of Advertising Pages between Reading Matter . . . . . . 382
International Alcoholic Congress . . . 123
Intermittent Fever and Atmospheric Temperature . . . . . . . . . . 240
Intestinal Anastomosis with Segmented Rubber Rings . . . . . . . 313
Instantaneous Cure of Whooping Cough . . . . . . . . . . . . . 173
Iodoform . . . . . . . . . . . 175
Medical Society of the State of New York . . . . . . . . . . . . . . 493
Mortality and Vital Statistics for the Eleventh Census . . . . . . . . . 122
Mission, The Fresh Air . . . . . . 757
Neuropathic Origin of Edema . . . . 312
New Health Law in Michigan . . . 242
New Lunacy Legislation . . . . . . 649
New York State Lunatic Asylum . . 444
Niagara University — Medical Department . . . . . . . . . . . . . 582, 647
Ourselves as Others See Us . . . . . 316
Physician and His Journals . . . . . 172
Preston Retreat . . . . . . . . . . 48
Proposed Reform in Medical Education . . . . . . . . . . . . . . 581
Protection Applied to the Practice of Medicine . . . . . . . . . . . . . 380
Rapid Transit for Physicians . . . . 496
Report of the Stevens' Commission . 495
Reform in Anatomical Nomenclature . 698
Rochester Infants' Summer Hospital . 123
Reflex Symptoms due to Phimosis . 170
Sanitary Organization of Women . . . 763
Street Car and the Public Health . . 46
'Twixt Tweedle-dum and Tweedle-dee . . . . . . . . . . . . . . . 763
Uniformity in Reprints . . . . . . . 172
Volume XXIX. . . . . . . . . . . 46
Volume XXIX.—Finis . . . . . . . 756

PAGE.

### Correspondence:

Foreign Letters: Crego . . . . 571, 642
Instantaneous Cure of Whooping Cough: Hartwig . . . . . . . . 237
Massey's Electricity in the Diseases of Women: Massey . . . . . . 37
Medical Department University of Vermont vs. The New York Post Graduate School: King. . . . . 693
Merritt H. Cash, Prize Essay . . . . 577

### Societies:

American Medical Association. . . . 584
American Public Health Association 178
American Society of Microscopists . . 768
American Association of Obstetricians and Gynecologists . . . . . . . . 768
Association of Railway Surgeons, Meeting of the National . . . . 768
Buffalo Medical and Surgical Association . . . 25, 91, 289, 366, 481, 626, 689
Buffalo Pathological Society, 99, 234, . . . . . . . . . . . . 417, 543, 688
Buffalo Obstetrical Society . . . . . 686
Buffalo Medical and Surgical Association . . . . . . . . . . . . . 744
International Congress for Study of Alcoholism. . . . . . . . . . . . 116
Medical Society of Erie County Proceedings . . . . . . . . . . . . . 733
New York Academy of Medicine — Section on Orthopedic Surgery 469, 679
Obstetrical Society of Philadelphia . . . . . . . . . . . . . . 229; 278, 431
Philadelphia County Medical Society . . . . . . . . . . . . . . . . 221, 599
Southern Surgical and Gynecological Association . . . . . . . . . . . 178
Tenth International Medical Congress . . . . . . . . . . . . . 178
The American Society of Microscopists . . . . . . . . . . . . . . . 50
The American Association of Obstetricians and Gynecologists . . . . 51
The American Pediatric Society . . . 123
The American Gynecological Society . 123
The American Association of Obstetricians and Gynecologists. . . . . 179
The American Gynecological Society . 179
The American Society of Microscopists. 124
The American Association of Obstetricians and Gynecologists . . . . . 124
The Association of American Physicians . . . . . . . . . . . . . . 123
The Buffalo Pathological Society, 292, 383
The Canadian Medical Association . . 179
The Congress of American Physicians and Surgeons . . . . . . . . . . . 246

PAGE.

The Eighty-fourth Annual Meeting of the Medical Society of the State of New York . . . . . . . . . . . . 383
The Forty-first Annual Meeting of the American Medical Association . . 499
The Iowa State Medical Society . . . 585
The Mississippi Valley Medical Society . . . . . . . . . . . . . . . 123
The Medical Society of the State of New York . . . . . . . . . . . 245
The Medical Society of Erie County, 383
The McDowell Medical Society . . . 246
The Medical Association of the State of Alabama . . . . . . . . . . . 585
The Medical Society of the State of New York . . . . . . . . . . . 447
The North of England Obstetrical and Gynecological Society . . . . 499
The National Association of Railway Surgeons . . . . . . . . . . . . 585
The Southern Surgical and Gynecological Association . . . . . 206, 316
The Tenessee State Medical Society . 582
Transactions of the American Association of Obstetricians and Gynocologists . . . . . . . . . . . . . 704
Transactions of the Buffalo Obstetrical Society . . . . . . . 478, 568
Tri-State Medical Association . . . . 177

### Obituary Record:

Byford, Dr. William H. . . . . . . 767
Coskery, Dr. Oscar J. . . . . . . . 52
Dimon, Dr. Theodore . . . . . . . 125
Harvey, Dr. Thomas B. . . . . . . 384
Howe, Dr. Joseph W. . . . . . . . 767
Hunter, Dr. James B. . . . . . . . 52
King, Dr. Thomas J. . . . . . . . . 318
McMillan, Dr. Charles . . . . . . . 445
Mott, Dr. Alexander B. . . . . . . 125
Prince, Dr. David . . . . . . . . . 445
Ricord, Professor . . . . . . . . . 246
Rochester, Mrs. Margaret Munro . . . 445
Sayre, M. D., Lewis Hall . . . . . . 446
Squire, Dr. Truman Hoffman . . . . 445
Strong, Dr. Phineas H. . . . . . . . 497
Taylor, Dr. Isaac E. . . . . . . . . 318
Volkmann, Professor Richard Von . . 384
Wilson, Dr. Ellwood . . . . . . . . 52
Wood, Dr. Charles S. . . . . . . . 498

### Personals:

Ayres, C. S., A. M., M. D. . . . . . 498
Banta, Dr. Rollin L. . . . . . . . . 177
Belfield, Dr. Wm. T. . . . . . . . . 712
Billings, Dr. Frank S. . . . . . . . 244
Cary, Dr. Charles . . . . . . . . . 177
Clark, Dr. Edward . . . . . . . . . 53
Clark, Dr. Charles P. . . . . . . . 245
Crego, Dr. F. S. . . . . . 498, 566, 701

PAGE.

Cushing, Dr. Clinton . . . . . . . . 586
Daniels, Dr. C. M. . . . . . . . . . 498
Dorland, Dr. E. T. . . . . . . . . . 245
Dornan, Mr. Wm. J. . . . . . . . . 592
Emmet, Dr. Bache McE. . . . . . . 318
Hanks, Dr. Horace F. . . . . . . . . 318
Gardner, U.S.A., Ass't Sur'n Edwin F. 318
Goldberg, Dr. Jacob . . . . . . . . 318
Granger, Dr. Wm. D. . . . . . . . . 701
Grant, Dr. H. Y. . . . . . . . . . . 177
Gray, Dr. John R. . . . . . . . . . 766
Green, Dr. S. S. . . . . . . . . . . 766
Gregory, Dr. W. G. . . . . . . . . . 53
Greene, Dr. Joseph C. . . . . . . . 245
Greene, Dr. W. D. . . . . . . . . . 447
Howe, Dr. L. . . . . . . . . . . . . 446
Jackson, Dr. T. D. . . . . . . . . . 711
Jessel, Mr. Henry K . . . . . . . . 317
Kibler, Dr. C. B. . . . . . . . . . . 585
Krauss, M. D., Wm. C. . 125, 177, 651, 766
Lewis, Dr. Daniel . . . . . . . . 446, 701
Long, Dr. Eli H. . . . . . . . . . . 766
Mallon, Mrs. Isabel . . . . . . . . . 770
Manton, Dr. W. P. . . . . . . . . . 244
Matthews, Dr. Joseph M. . . . . . . 766
Maus, U.S.A., Ass't Surgeon Louis M. 318
McPhatter, Dr. Neil L. . . . . . . . 766
McPherson, Dr. G. W. . . . . . . . . 446
McMurtry, Dr. L. S. . . . . . . . . 498
Miller, Dr. John A. . . . . . . . . . 179
Park, Dr. Roswell . . . . . . . . . . 585
Parmenter, Dr. John . . . . . . . . 52
Parrish, Dr. William H. . . . . . . . 766
Potter, Dr. William Warren . . . . . 53
Price, Dr. Joseph . . . . . . . . . . 125
Renner, Dr. W. Scott . . . . . . . . 701
Raum, General Green B. . . . . . . . 700
Rohé, Dr. George H. . . . . . . . . 701
Roberts, Dr. John B. . . . . . . . . 177
Ross, Dr. T. Haven . . . . . . . . . 701
Smith, Dr. J. Lewis . . . . . . . . . 712
Stockton, Dr. Charles G. . . . . . . 177
Tremaine, U. S. A., Surgeon William S. . . . . . . . . . . . 177, 318
Wall, Dr. Charles A. . . . . . . . . 766
Wetmore, Dr. George T. . . . . . . . 766
Widner, Dr. Joe. Elmer . . . . . . . 498
York, Dr. G. W. . . . . . . . . . . 498

### Reviews:

Agnew: Principles and Practice of Surgery . . . . . . . . . . . . . 770
Aitken: On the Animal Alkaloids . 589
Adams: Thomas Jefferson and the University of Virginia . . . . . . 129
American Gynecological Society Transactions . . . . . . . . . . . . . . 58
A Treatise on Diseases of the Nose and Throat. By Francke Huntington Bosworth, A. M., M. D. . . . 449

PAGE.

Attfield: Chemistry: General, Medical and Pharmaceutical . . . . . 385
Bacigalupi: Immunity Through Leucomaines . . . . . . . . . . . 56
Bartley: Text-Book of Medical Chemistry . . . . . . . . . . . 505
Billings, J. S.: National Medical Dictionary . . . . . . . . . . . . . 504
Billings, F. S.: Original Investigatton of Cattle Diseases in Nebraska . . . 323
Billings, J. S.: Index Catalogue of the Library of the Sur geon-General's Office United States Army . . . 386
Bradford: A Treatise on Orthopedic Surgery . . . . . . . . . . . 774
Brubaker: A Compend of the Human Physiology . . . . . . . . . . . 776
Buck: A Reference Hand-Book of the Medical Sciences . . . . . . . 324
Caird and Cathcart: A Surgical Hand-Book for the Use of Practitioners and Students . . . . . . 325
Cathell: Book on the Physician Himself and Things that Concern His Reputation and Success. . . . 132
Clevenger: Spinal Concussion . . . 651
Cragin: Essentials of Gynecology . . 775
Davenport: Diseases of Women . . . 185
Delafield: A Hand-Book of Pathology, Anatomy and Histology . . 502
Erlenmyer: — Treatment of Morphia Disease . . . . . . . . . . . . 60
Fox: Photographic Illustrations of Skin Diseases. . . . . . . . . . 253
Goodhart-Starr: A Guide to the Diseases of Children . . . . . . . . 589
Greene: An Introduction to Pathology and Morbid Anatomy . . . 251
Hirst: A System of Obstetrics by American Authors . . . . . . . . 126
Holland: — The Urine, The Common Poisons, and The Milk . . . . . . 385
Hypnotism: Its History and Present Development. By Fredrick Björnström, M. D. . . . . . . . 453
Ireland: Through the Ivory Gate . 500
Jacobi, M. P.: Physiological Notes on Primary Education and the Study of Language . . . . . . . . . . 61
Kerr: Inebriety — Its Etiology, Pathology, Treatment and Jurisprudence 188
Keating: Cyclopedia of the Diseases of Children . . . . . . . . . 53, 246
King: A Manual of Obstetrics . . . 225
Kuhn: L'Enseignement et L'Organization de L'Art Dentaire aux E'tats Unis . . . . . . . . . . . . . . 186
Landois-Sterling: A Text-Book of Human Physiology . . . . . . . . 508
Liebig-Rohé: Practical Electricity in Medicine and Surgery . . . . . 703
Mills: A Text-Book of An mal Physiology . . . . . . . . . . . . . . 319
Maisch: A Manual of Organic Materia Medica . . . . . . . . . . 653
Nissen: Swedish Movement and Massage Treatment . . . . . . . 57
Morris: Essentials of Materia Medica . 386
Noyes: A Text-Book on Diseases of the Eye . . . . . . . . . . . . 702
Norris: Syllabus of the Obstetrical Lectures in the Medical Department of the University of Pennsylvania . . . . . . . . . . . . . 652
Parvin: Lectures on Obstetric Nursing . . . . . . . . . . . . . . . 253
Physician's Hand-Book for 1890 . . . 453
Playfair: A Treatise on the Science and Practice of Midwifery . . . . 252
Potter: Hand-Book of Materia Medica, Pharmacy, and Therapeutics . . . . . . . . . . . . . . 705
Price and Eagleton: The Nervo-Vascular Syst m. . . . . . . . . . 254
Proceedings of the American Society of Microscopists . . . . . . . . . 703
Prudden: The Story of Bacteria and their Relation to Health and Disease . . . . . . . . . . . . . . . 326
Rawlinson: The Story of Phœnicia . 60
Roberts: Cure of Crooked and Deformed Noses . . . . . . . . . . . 590
Sajous: Annual of the Universal Medical Sciences . . . . . . . . . 187
Saundby: Lecture on Bright's Disease . . . . . . . . . . . . . . . 61
Schmidt-Rimpler, Roosa: Ophthalmology and Ophthalmoscopy, for Practitioners and Students of Medicine . . . . . . . . . . . . . . 246
Schultze: Lehrbuck der Hebammenkunst . . . . . . . . . . . . . 588
Semple: Essentials of Pathology and Morbid Anatomy . . . . . . 186
Shoemaker-Aulde: A Treatise on Materia Medica, Pharmacology, and Therapeuties . . . . . . . . . . . 507
Sixteenth Annual Report of the State Commissioner in Lunacy for the Year 1888 . . . . . . . . . . . 62
Skene: Education and Culture as Related to the Health and Diseases of Women . . . . . . . . . . . . . 509
Smith: Diabetes, Mellitus and Insipidus . . . . . . . . . . . . . . 509
Smith: The Physiology of the Domestic Animals . . . . . . . . . 131
Society Transactions . . . . . . . . 58
Stelwagon: Essentials of Diseases of the Skin . . . . . . . . . . . . 775

PAGE.

Symonds: A Manual of Chemistry, for the Use of Medical Students . . 387

Tait: Diseases of Women and Abdominal Surgery . . . . . . . . 771

Transactions of the American Surgical Association . . . . . . . . 180

Transactions Medical Society, S. N. Y. 58

Transactions New York State Medical Association . . . . . . . . . . . 58

Ultzman: The Neuroses of the Genito-Urinary System in the Male 775

Valk: Errors of Refraction . . . . 183

Witthaus: A Labaratory Guide in Urinalysis and Toxicology . . . . 133

Young: Synopsis of Human Anatomy . . . . . . . . . . . . . . . 59

Williams: The International Medical Annual . . . . . . . . . . . 653

Winckel-Edgar: A Text-Book of Obstetrics . . . . . . . . . . . . 587

Winckel: A Hand-Book of Diseases of Women . . . . . . . . . . . . 775

Worcester: Monthly Nursing . . . 588

Wood's Medical and Surgical Monographs . 59, 183, 188, 250, 322, 506, 586 . . . . . . . . . . . . . . . . . 773

Woodbury: On Disordered Digestion and Dyspepsia . . . . . . . . . 189

Wood: Syphilis of the Nervous System . . . . . . . . . . . . . . . 189

PAGE.

**Journalistic and Literary:**

Valuable and Unique Business Calendar . . . . . . . . . . . . . . . 389
Epitome of Examination of Recruits . 711
Belford's Magazine . . . . . . . 456
Best Serial Stories. . . . . . . . . 256
Buffalo Sunday Express, . . . . . . 175
Dietetic Gazette . . . . . . . . . . 712
Gould's Medical Dictionary . . . . 592
Holiday Number of the Trained Nurse 456
Kilmer's Physician's Pocket Ledger . 192
Maryland Medical Journal . . . . . 711
Progress of Inventions since 1845 . . . 191
Pronunciation of Gynecology . . . . 592
St. Louis Polyclinic . . . . . . . . . 444
The Illustrated American . . . . . 769
The American Journal of Medical Sciences . . . . . . . . . . . . . 769
The Annals of Gynecology and Pediatry . . . . . . . . . . . . . . . 769
The Professional Canvasser . . . . . 64
The Open Court . . . . . . . . . . 64
The Transatlantic . . . . . 256, 390, 592
The Microscope . . . . . . . . . . 256
The Medical Mirror . . . . . . . . 328
The Philadelphia Medical and Surgical Reporter . . . . . . . . . . 390
The National Magazine . . . . . . 391
The Dixie Doctor . . . . . . . . . . 444
The Cosmopolitan. . . . . 389, 512, 591
The North Carolina Medical Journal. 456
The Detroit Free Press Souvenir . . . 456
Transactions of Special Societies, 1889. 592
Transactions of the American Association of Obstetricians and Gynecologists . . . . . . . . . . . . . . . 328

### Society Meetings.

THE AMERICAN SOCIETY OF MICROSCOPISTS will hold its meeting for the current year in the Buffalo Library Building, in this city, August 20th, 21st, 22d, and 23d instant. The active preparations now making by the Buffalo Microscopical Club for the reception of the visitors, presages a large and successful meeting. Let all our citizens take pride in entertaining this distinguished body of scientific men and women.

THE AMERICAN ASSOCIATION OF OBSTETRICIANS AND GYNECOLOGISTS will hold it next annual meeting at the Burnet House, Cincinnati, O., in the rooms lately occupied by the Military Order of the Loyal Legion, on Tuesday, Wednesday, and Thursday, September 17, 18, and 19, 1889. No formal invitations will be issued to non-members, but the Association hereby extends a cordial invitation to such members of the profession wherever resident as may feel interested, to attend the meeting and participate in the proceedings. The papers and discussions will embrace subjects pertaining to obstetrics, gynecology, and abdominal surgery.

By order of the President.

WILLIAM WARREN POTTER, *Secretary.*

---

### Medical College Notes.

### BOOKS AND PAMPHLETS RECEIVED.

A System of Obstetrics by American Authors. Edited by Barton Cooke Hirst, M.D. Vol. II. Illustrated with 221 engravings on wood. Philadelphia: Lea Brothers and Co. 1889.

Diseases of Women: A Manual of Non-surgical Gynecology, designed especially for the use of Students and General Practitioners. By F. H. Davenport, A. B., M. D. With numerous illustrations. Philadelphia: Lea Brothers & Co. 1889.

The Physician Himself, and Things that Concern His Reputation and Success. By D. W. Cathell, M. D., Baltimore, Md. The ninth edition, revised and enlarged. Philadelphia and London: F. A. Davis. 1889.

Bureau of Education. Contributions to American Educational History. Edited by Herbert B. Adams. No. 3. The History of Education in North Carolina, by Charles Lee Smith. No. 4. History of Higher Education in South Carolina, with a Sketch of the Free-School System, by Colyer Meriwether, A. B. No. 5. Education in Georgia, by Charles Edgeworth Jones. No. 6. History of Education in Florida by George Gary Bush, Ph. D. No. 7. Higher Education in Wisconsin, by Wm. F. Allen and David E. Spencer.

"Curorte" enthaltend: die wichtigsten Bada- und Cur-Anstalten Mittel-Europa's nebst Biographien und Portrats hervorragender Aerzte. Bei Dr. Adolf Kallay. Wein, 1889. Verlag von Wilhelm Braumuller u. Sohn, k. k. Hof- und Universitats-Buchhandlung.

Report of a Case of Stricture of the Rectum, the Probable Result of a Specific Vaginitis. By Lewis H. Adler, Jr., M. D., Philadelphia. Reprint from the *Medical and Surgical Reporter*, June 29, 1889.

A Case of Hodgkin's Disease, accompanied with a Possible Resulting Paraplegia. Reported by Lewis H. Adler, Jr., M. D., Philadelphia. Reprint from the *Medical News*, January 12, 1889.

A Year's Work in Abdominal Surgery. By Clinton Cushing, M. D., San Francisco, Cal. Reprint from *Pacific Medical Journal*, July, 1889.

Report of Amputations performed at the Hospital of the University of Pennsylvania from September 30, 1874, to December 31, 1888. By Lewis H. Adler, Jr., M. D. Reprint from the *Medical and Surgical Reporter*, May 11, 1889.

Report of a Case of Hystero-Epilepsy in a Man. By Lewis H. Adler, Jr., M.D. Reprint from the *Medical News*, March 9, 1889.

Hernia. A Comparison of the Various Methods Adopted for its Cure; Inviting Discussion of their Respective Merits. By C. H. Mastin, M. D., LL.D., (University of Pennsylvania,) of Mobile, Ala. Reprint from the Transactions American Surgical Association. 1889.

Secondary Mixed Infection in Some of the Acute Infectious Diseases of Children. A Thesis read before the Chicago Gynecological Society, January 25, 1889. By Bayard Holmes, M. D. Reprint from the *North American Practitioner*, February, March and June, 1889.

Semi-Annual Price List of Standard Pharmaceutical Preparations and Pharmacopeial Reagents. Manufactured by E. R. Squibb, Brooklyn, N. Y. Revised July 1, 1889.

Seventh Annual Announcement of the Medical Department of Niagara University, Buffalo, N. Y. Session of 1889–'90.

University Medical College of Kansas City. Formerly Medical Department of the University of Kansas City. Ninth Annual Announcement and Catalogue of Session, 1888–'89. Kansas City, Mo.

Forty-fourth Annual Announcement of the Medical Department of the University of Buffalo for the Session of 1889–'90, with Catalogues of previous Session. Buffalo.

Annual Announcement and Catalogue of the Baltimore Medical College, Baltimore, Md. Session 1889–'90.

Annual Announcement and Catalogue of the College of Physicians and Surgeons, Baltimore, Md., 1889–'90.

Annual Catalogue and Announcement of the Western Pennsylvania Medical College, Pittsburgh. Sessions of 1889–'90.

Twenty-ninth Annual Announcement of the Bellevue Hospital Medical College, 1889–'90, with the list of graduates for 1889.

Monthly Bulletin of the New York State Board of Health, May, 1889.

Health in Michigan, June, 1889.

Report of Deaths and Contagious Diseases for the month of June, 1889. Newport, R. I.

Weekly Abstract of Sanitary Reports. Vol. IV. Nos. 27, 28, 29, 30.

### Literary and Other Notes.

The Professional Canvasser is an advertising medium as well as a supplement to The Professional Reference Lists, which is an elaborate price list, presenting the manufactures of leading American and European firms.

The Professional Canvasser is published at irregular dates. Each issue combines subjects of interest to the medical profession, and the several issues are as diverse as the interest represented is complex.

The copy last received is a twenty-page pamphlet, combining price lists and specimen plates of engraving, stamping, blocking, embossing, illuminating, etc.

Arrangements have been completed to supply copies to one hundred thousand applicants, but each copy costs over ten cents, and applicants are solicited to remit that amount to cover costs of postage, etc.

Address all communications to Fred. D. Van Horen, 23 Clinton place, New York.

---

Mr. George John Romanes, the distinguished author of Mental Evolution in Man: Origin of Human Faculty, has contributed to *The Open Court* of July 11th, an article entitled "The Psychic Life of Microöganisms." The public will recall M. Binet's able series of essays in Volume II. of *The Open Court*, in which the soul-life of these tiny and interesting beings was so carefully discussed. The essays were afterwards published in book form by The Open Court Publishing Co. In a preface written especially for the American edition, M. Binet took issue with Mr. Romanes relative to the stage in animal development at which psychological powers first appear. The criticism has attracted much attention. The eminent English scientist, in turn, now replies to the strictures of the French *savant*. The controversy will be of interest to all. To those who have read M. Binet's monograph, the reply of Mr. Romanes will be found an appropriate supplement.

---

The Private Hospital for Rectal Diseases, recently opened by Dr. Edward Clark, in this city, affords a most excellent opportunity for those afflicted in this way to receive that special treatment and skilful care so absolutely necessary to perfect recovery.

---

Good.—Judge Andrews, of New York, has lately decided that a stable, however well it may be built, is a nuisance in the vicinity of handsome residences, and that an action can be brought to have it removed.—*Sanitary Era*, July 15th.

